# Increased affinity by dimerization of radiolabeled Affibody molecule ATH001 targeting PDGFRβ

**DOI:** 10.1186/s41181-026-00439-x

**Published:** 2026-03-16

**Authors:** Ayman Abouzayed, Hugo Olsson, Natalia Papadopoulos, Bogdan Mitran, Hemantha Mallapura, Tahmida Akter, Irina Velikyan, Olle Korsgren, Carl-Henrik Heldin, John Löfblom, Michael Wagner, Olof Eriksson

**Affiliations:** 1 Science for Life Laboratory, Department of Medicinal Chemistry, Uppsala University, Dag Hammarskjölds väg 14C, 3tr, 751 83 Uppsala, Sweden; 2 Antaros Tracer AB, Uppsala, Sweden; 3 Division of Protein Engineering, Department of Protein Science, KTH Royal Institute of Technology, Stockholm, Sweden; 4 SciLifeLab, Department of Medical Biochemistry and Microbiology, Uppsala University, Uppsala, Sweden; 5 PET Center, Akademiska Sjukhuset, Uppsala, Sweden; 6 Department of Immunology, Genetics and Pathology, Uppsala University, Uppsala, Sweden

**Keywords:** Platelet-derived growth factor receptor, Dimer, PET, Radioligand therapy, Affibody

## Abstract

**Background:**

Platelet-derived growth factor receptor–β (PDGFRβ) is a canonical marker of pericytes and stromal cells in most tissues. It is present on cancer associated fibroblasts (CAFs) in the tumor microenvironment and has thus been proposed as a potential therapeutic target both for anti-cancer drugs as well as for radioligand therapy (RLT). ATH001 is a novel Affibody molecule-based radiopharmaceutical in clinical development for targeting of PDGFRβ. We hypothesize that dimerization of ATH001 could improve affinity to PDGFRβ, leading to better characteristics for in vivo targeting. The dimeric construct, named ATH022, was generated by conjugation of two chemically synthesized ATH001 binders to a tri-functional linker comprising a DOTA chelator. Here, we present a comprehensive in vitro and in vivo evaluation of Gallium-68 and Indium-111 labeled ATH022, in direct comparison with ATH001.

**Results:**

DOTA-ATH022 acted as a PDGFRβ antagonist and competed dose-dependently with the endogenous ligand PDGF-BB. Affinity of the dimer was improved approximately tenfold compared to DOTA-ATH001, mainly due to strongly decreased off-rate. Radiolabeled DOTA-ATH022 demonstrated higher specific binding and longer retention to U87 cells in vitro. Radiolabeled DOTA-ATH022 also exhibited elevated binding in PDGFRβ-positive tissues spleen and U87 tumors, that could be blocked by ATH001 in excess. Binding of DOTA-ATH022 in PDGFRβ avid and well-perfused spleen was 3 times higher than for DOTA-ATH001, but tumor binding was lower. In vivo retention in spleen was 5 times longer for Indium-111 labeled ATH022, in agreement with its in vitro binding characteristics.

**Conclusions:**

DOTA-ATH022 is a novel dimerized version of ATH001, with significantly improved affinity towards PDGFRβ. Retention of radiolabeled DOTA-ATH022 in PDGFRβ-avid tissues and tumors was increased, however uptake in solid tumor tissue was not improved.

**Supplementary Information:**

The online version contains supplementary material available at 10.1186/s41181-026-00439-x.

## Introduction

Platelet-derived growth factor receptor-β (PDGFRβ) is a canonical marker of pericytes and stromal cells in most tissues. It is involved in vascularization and angiogenesis, and is associated with fibrotic pathology and tissue remodeling (Heldin and Westermark 1999). PDGFRβ is also present on cancer associated fibroblasts (CAFs) in the tumor microenvironment and has thus been proposed as a potential therapeutic target, both for anti-cancer drugs as well as for radioligand therapy (RLT) (Cords et al. 2023).

**Fig. 1:**
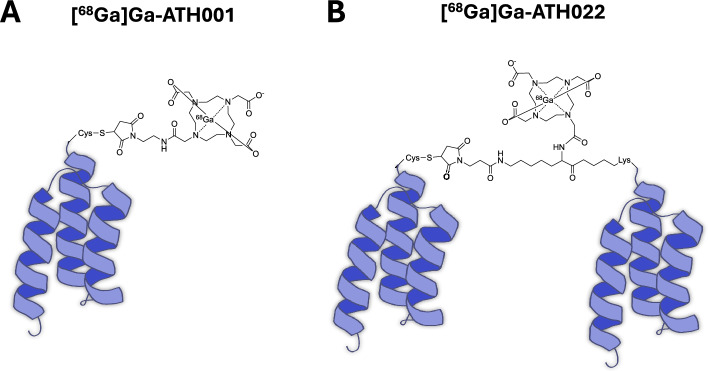
Schematic structure of the original tracer construct [^68^Ga]Ga-ATH001 (**A**) and the dimerized [^68^Ga]Ga-ATH022, using a novel trimodal linker with two handles for binding molecules (2 × ATH001) and one for radiolabeling (e.g. Galllium-68)

ATH001 (previously referred to Z_09591_ or Z_PDGFRβ_ in the literature) is an Affibody molecule developed for molecular imaging of PDGFRβ (Lindborg et al. 2011; Wegrzyniak et al. 2023, 2024, 2025). ATH001 demonstrates sub-nanomolar affinity to human recombinant PDGFRβ, combined with a relatively small molecular size (6.5 kDa) allowing for rapid clearance and tissue penetration. [^68^Ga]Ga-DOTA-Cys-ATH001 (here abbreviated as [^68^Ga]Ga-ATH001 or monomeric ATH001, Fig. 1A) is in clinical development as a positron emission tomography (PET) radiopharmaceutical for molecular imaging of PDGFRβ in fibrotic disease and cancer (NCT06562361, NCT06956560) (Yashaswini et al. 2025; Velikyan et al. 2025). ATH001 binds to the extracellular domain of human and murine PDGFRβ with picomolar and nanomolar affinity, respectively, and acts as an antagonist by competing with the PDGF-BB ligand for binding to PDGFRβ (Yashaswini et al. 2025).

Target affinity is critically important for any theranostic PET or RLT radiopharmaceutical, and correlates to the magnitude of binding, tissue exposure and time-on-target. Thus, rational strategies for improved affinity should be employed to optimize radiopharmaceutical performance, while being constrained by preserving other characteristics of the Affibody molecule scaffold such as small size and rapid biodistribution.

Since PDGFRβ forms homodimers during the process of binding and activation by PDGF-BB, it is reasonable to assume that PDGFRβ proteins are present in close proximity at the cell surface, in particular during activation. We therefore hypothesized that dimerization of ATH001 would improve the affinity towards PDGFRβ through avidity. A novel modular trifunctional linker construct was used to generate a construct incorporating two ATH001 Affibody molecules together with a radiometal chelator (termed ATH022) (Fig. 1B).

Here, we present a full characterization of ATH022 in vitro and in vivo in a relevant cancer model, in direct comparison with monomeric ATH001.

## Material and methods

### Affibody molecule generation

ATH001 monomeric Affibody molecules were produced by custom solid phase peptide synthesis (SPPS) using standard manufacturing strategies and commercially available reagents (Almac). DOTA-ATH001 was generated by insertion of a unique cysteine at the C-terminal, where a 2,2′,2′′,2′′′-(1,4,7,10-Tetraazacyclododecane-1,4,7,10-tetrayl)tetraacetic acid (DOTA) chelator was added via maleimide chemistry (Fig. 1A).

For the dimeric construct, ATH001 was conjugated to a tri-functional linker via an orthogonally protected lysine residue at the C-terminal. The tri-functional linker further contained handles for a DOTA chelator, as well as a maleimide group for further modifications, where a second ATH001 Affibody molecule with a cysteine residue at the C-terminal was added. The final construct ATH001-linker (DOTA)-ATH001, was termed ATH022 (Fig. 1B).

DOTA-ATH001 (7063.8 Da) and DOTA-ATH022 (13,765.5 Da) were evaluated for purity (> 95% as assessed by Reversed-phase-high-performance liquid chromatography (RP-HPLC)) (Supplementary Fig. 1A, B) and identity (GC–MS) (Supplementary Fig. 1C, D) before lyophilization and vialling in 200 µg aliquots.

### Surface plasmon resonance assay

Parallel channels on a Series S sensor chip CM5 (Cytiva, cat.no. 29149603) were immobilized with carrier-free recombinant human PDGFRβ-Fc (R&D Systems, cat.no. 385-PR-100/CF) dissolved in pH 5.0 sodium acetate at 25 μg/mL concentration. Using an NHS chemistry-based coupling kit (Cytiva, cat.no. BR100050), the ligand was attached to a level of 1000 response units (RU) on a Biacore 8 K instrument. Across three separate multi-cycle kinetics assessments, DOTA-ATH001 or DOTA-ATH022 were injected over the respective channels at 25 °C. A contact time of 120 s was followed by 600 s dissociation using PBS-T (PBS with 0.1% Tween20) as running buffer; the chip was regenerated with 10 mM NaOH 1 M NaCl solution between each concentration. Six concentrations ranging between 0.625 and 20 nM were used for determination of kinetic parameters from each multi-cycle experiment, fitting a 1:1 interaction model to the obtained sensorgrams.

### Cell culture

PDGFRβ expressing human glioma cell line U87 and human hepatic stellate cell line LX-2 were used for the in vitro binding experiments. U87 cells were obtained from ATCC (US). The U87 cells were cultured in DMEM/F12 medium (Biowest) enriched with 10% fetal bovine serum (FBS; Sigma Aldrich) and a combination of antibiotics containing 10,000 U/ml penicillin and 10,000 µg/ml streptomycin (Gibco, USA).

The human hepatic stellate cell line LX-2 (SCC064, Sigma) was maintained under similar conditions but with a reduced FBS concentration of 2% to optimize cell-specific growth and maintenance requirements. Since both cell types are adherent, 0.25% trypsin–EDTA (Gibco, UK) was used for cell detachment. All cultures were incubated at 37 °C in a controlled atmosphere containing 5% CO₂.

### PDGFRβ activation assay

LX-2 cells were starved overnight in DMEM media containing 0.1% FBS. Cells were treated with starvation media containing 5 ng/ml PDGF-BB with or without increasing concentrations of ATH022 and incubated for 20 min in order to allow ligand-induced dimerization and transactivation of PDGFRβ. A separate sample was treated with 200 ng/ml of ATH022 in the absence of PDGF-BB as a negative control. Cells were washed twice in ice-cold PBS and lysed in RIPA buffer (0.5% deoxycholate, 0.1% SDS, 1% Triton X-100, 10% glycerol, 20 mM Tris, pH 7.4, 150 mM NaCl), supplemented with Halt protease and phosphatase inhibitors cocktail (ThermoScientific). Cell debries were removed by centrifugation at 11 000 g for 15 min and PDGFRβ was immunoprecipitated from the supernatant at 4 °C overnight using ctβ antibody (Hasumi et al. 2007). Immunocomplexes were captured using Rec-protein G-sepharose 4B conjugate (ThermoScientific) and eluted with the RIPA lysis buffer, containing 1% SDS. Eluates containing immunoprecipitated PDGFRβ were resolved on SDS-polyacrilamide gel electrophoresis (SDS-PAGE), electrotransferred to PVDF membrane (Immobilon) and immunoblotted using antibody against total PDGFRβ (AF385, R&D) or Tyr857 phosphorylated PDGFRβ (Cell Signaling). The secondary antibodies were HRP-conjugated anti-goat IgG (ThermoScientific) and anti-rabbit IgG (Jackson Immunoresearch), respectively. The proteins were visualized with the chemiluminescent detection system SuperSignal West Dura Extended Duration Substrate (34,075, ThermoScientific) on a charge-coupled device (CCD) camera (BioRad).

### Gallium-68 and Indium-111 radiolabeling of ATH001 and ATH022

[^68^Ga]GaCl_3_ (t_1/2_ = 68 min) labeled ATH001 and ATH022 were subjected to in vitro binding specificity analysis and in vivo study. ATH001 or ATH022 labeled with [^111^In]InCl_3_ (t_1/2_ = 67.2 h) was used for LigandTracer assays as well as for some in vivo experiments where a longer observation was required.

[^68^Ga]GaCl_3_ was obtained by elution of an IGG100 ^68^Ge/^68^Ga generator (Eckert & Ziegler, Germany) with 0.1 M metal-free HCl. DOTA-ATH001 or DOTA-ATH022 were radiolabeled with ^68^Ga by adding 20 or 40 μl of the precursor (1 µg/µl) to 200–300 μl of 1 M sodium acetate buffer (pH 4.65). The mixture was incubated with 200–300 μl of ^68^Ga-eluate (100 – 190 MBq, at end of radiolabeling) for 15 min at 75°C. After incubation, [^68^Ga]Ga-ATH001 and [^68^Ga]Ga-ATH022 were purified using NAP-5 size-exclusion columns and the purity was determined by RP-HPLC.

For radiolabeling of DOTA-ATH001 or DOTA-ATH022 with ^111^In, 20–28 μl (1 µg/µl) of the precursor was mixed with 60–80 μl 0.2 M sodium acetate buffer (pH 5.5) and radiolabeled with ^111^In (40–50 MBq, Curium Pharma). The reaction mixture was incubated at 75 °C for 30 min. The radiochemical purity was analyzed using RP-HPLC. The Indium-111 radiolabeled constructs were termed [^111^In]In-ATH001 and [^111^In]In-ATH022.

### Lipophilicity assay

The lipophilicity of [^68^Ga]Ga-ATH001 and [^68^Ga]Ga-ATH022 was determined using a modified shake-flask method. A mixture of 1 mL, consisting of 500 µL of n-octanol and 500 µL of phosphate-buffered saline (PBS, pH 7.4), was prepared in a 1.5 mL low protein-binding centrifuge tube. A tracer sample (20–50 µL) was added to the tube, and reference tubes were prepared with the same volume of the tracer to serve as a standard for normalization. The sample tubes were vortexed for 1 min to ensure thorough mixing, followed by brief centrifugation at high speed (20–30 s) to achieve phase separation. The bottom aqueous layer (PBS, 500 µL) was carefully withdrawn using a micropipette. Aliquots (100 µL) from the PBS and octanol phases from each cycle were collected, and the radioactivity in all samples including the reference were measured using a Wizard gamma counter. The lipophilicity (Log P) was calculated as the logarithm (base 10) of the ratio of counts in the organic phase to those in the aqueous phase, normalized to the activity of the reference sample.

### Plasma stability of [^68^Ga]Ga-ATH001 and [^68^Ga]Ga-ATH022

The stability of the radiolabeled tracers ([^68^Ga]Ga-ATH001 and [^68^Ga]Ga-ATH022) in human plasma was assessed using a standardized protocol. The human plasma sample was mixed with the radiolabeled compound at a 9:1 volume ratio (typically 450 µL plasma and 50 µL tracer solution) in a low-protein-binding 1.5 mL Eppendorf tube and vortexed thoroughly to ensure homogeneous mixing. After incubated at 37 °C for 60 min to simulate physiological conditions, plasma proteins were precipitated by the addition of methanol in a 2:1 (v/v) ratio relative to the total plasma volume. The mixture was vortexed for proper mixing and precipitation. Subsequently, the sample was centrifuged at 10,000 rpm for 4–5 min at 4 °C. The supernatant and pellet (precipitate) fractions were carefully separated and measured for radioactivity using a calibrated gamma counter/dose calibrator. Then, the supernatant was filtered twice through a 0.22 µm nylon membrane to remove residual protein. Finally, an aliquot of the filtered supernatant (5–10 µL) was injected into a reverse-phase high-performance liquid chromatography (RP-HPLC) system to determine the radiochemical purity (RCP) of the soluble tracer fraction. By quantifying the radioactivity in both the precipitate and supernatant, and evaluating the RCP, the in vitro human plasma stability of the radiolabeled tracers was assessed.

### In vitro cell specificity and retention assay

U87 or LX-2 cells were seeded at a density of 5 × 10^5^ cells/ml culture media in 6-well plates and incubated at 37 °C with 5% CO₂. For the in vitro binding specificity assay, the U87 or LX-2 cells were incubated with 0.5 nM of Gallium-68 or Indium-111 radiolabeled ATH001 or ATH022 at 37 °C in 5% CO_2_ for an hour. For blocking, some of the cells were co-incubated with 16 µM of ATH001 (after 10 min of pre-incubation to allow receptor saturation). Following incubation, the cells were washed and trypsin–EDTA (0.25%) was added to detach the cells, after which the cells were collected and measured for radioactivity using a 2480 Wizard^2^automatic gamma counter (PerkinElmer, USA).

Additionally, retention of Indium-111 radiolabeled ATH001 or ATH022 was investigated in U87 cells. After 1 h incubation with [^111^In]In-ATH001 or [^111^In]In-ATH022, the cells were washed and replenished with fresh culturing media. The cells were cultured for 1 or 24 h, after which the remaining cell-bound radioactivity was measured. All individual experiments included triplicates for each condition.

### LigandTracer affinity assay

The affinity and binding kinetics of [^111^In]In-ATH001 and [^111^In]In-ATH022 towards PDGFRβ were evaluated using the LigandTracer Yellow instrument (Ridgeview Instruments AB, Sweden). U87 cells (3 × 10^6^ cells/dish) were seeded in a defined area of a petri-dish at least 24 h before the experiment. Initially, a baseline signal was measured for 30 min after which the radiolabeled ligand was added at increasing concentrations. For each concentration of tracer, the binding was measured (1 measurement/ second) until it reached a plateau. The association curves were established using Indium-111 tracers at concentrations of 0.1 and 0.3 nM for [^111^In]In-ATH022 or 0.3, 0.5 and 1 nM for [^111^In]In-ATH001. Following the association phases, the radioactive medium was removed and replaced with non-radioactive medium. The dissociation was measured for several hours until deemed complete. The kinetic data were analyzed using the TracerDrawer software using 1:1 binding kinetics (Ridgeview Instruments AB, Uppsala, Sweden).

### Animal handling and housing

Mice (Taconic) were housed in groups of five animals in ventilated cages, lined with bedding (GLP Aspen Bedding, TAPVEI). The animals had free access to food and water. Mice were housed at a constant temperature (22 °C) and humidity (50%) in a 12 h light/12 h dark cycle. The pigs were transported from a local breeder on the day of each experiment. All experimental protocols were approved by the Animal Ethics Committee of the Swedish Animal Welfare Agency. The procedures were performed in accordance with ARRIVE (Animal Research: Reporting of In Vivo Experiments) recommendations and Uppsala University guidelines on animal use (UFV 2007/724).

### Gallium-68 PET study in U87 xenografted mice

First, we evaluated the short-term in vivo biodistribution and tumor binding of [^68^Ga]Ga-ATH001 or [^68^Ga]Ga-ATH022, for up to two hours. Immunodeficient BALB/c nu/nu mice (female, 7–8 weeks, 20–25 g, *n* = 27) were used for the study (see Supplementary Fig. 2 for details). The mice were xenografted with PDGFRβ-expressing U87 cells, by subcutaneous implantation of 5 million cells (suspended in 50-80 µl growth medium) in the flank. Approximately 4 weeks after the implantation the tumors were of suitable size for the imaging study (≈0.5 cm^3^). Mice were injected intravenously with 1 μg of [^68^Ga]Ga-ATH001 (target dose of 2 MBq, corresponding to a molar dose of 0.15 nmol) (*n* = 10) or 2 μg (target dose of 2 MBq) of [^68^Ga]Ga-ATH022 (target dose of 2 MBq, corresponding to a molar dose of 0.15 nmol) (*n* = 17). The injected mass doses are based on previous dose-escalation studies demonstrating low or negligible mass effects or self-blocking at doses below 1 µg in mice for DOTA-ATH001 (Yashaswini et al. 2025). The peptide mass dose for dimeric DOTA-ATH022 was 2 µg to account for the almost double molecular weight and thus allow for direct comparison at the same injected molar mass.

Biodistribution was assessed at 1 or 2 h after the injection (*n* = 4 per group for the monomer or *n* = 5 for the dimer) by ex vivo organ distribution and gamma counter measurement.

A separate group of mice was co-injected with unlabeled 1 mg/kg ATH001 in excess to saturate the PDGFRβ before injection of [^68^Ga]Ga-ATH022 (*n* = 5) to demonstrate receptor binding specificity.

Additional mice with U87 xenografts (n = 5) were examined by in vivo PET (NanoPET/MRI, Mediso), after injection of 4 MBq [^68^Ga]Ga-ATH001 (*n* = 1 tracer alone and *n* = 1 co-administration of 1 mg/kg ATH001, corresponding to 2 µg or 0.3 nmol peptide) or [^68^Ga]Ga-ATH022 (*n* = 2 tracer alone and *n* = 1 co-administration of 1 mg/kg ATH001, corresponding to 4 µg or 0.3 nmol peptide). The animals were anaesthetized by sevoflurane and examined by a 15-min static PET scan after 1 h ([^68^Ga]Ga-ATH001 and [^68^Ga]Ga-ATH022) or after 2 h ([^68^Ga]Ga-ATH022 only).

The mice were euthanized by cervical dislocation, and the organs were collected post-mortem in vials to measure the radioactivity. After weighing, the radioactivity in each of the organs and tissues were measured in a well counter. All the radioactive data was decay corrected, and the in vivo uptake was reported as injected dose per gram of tissue (%ID/g) corrected for both for injected dose and tissue weight.

### Indium-111 biodistribution study in U87 xenografted mice

To examine the uptake and long-term retention of DOTA-ATH001 and DOTA-ATH022 in U87 tumor and other tissues, additional groups of Balb/c nu/nu xenografted mice (female, 7–8 weeks, 20-25 g, *n* = 40) were administered [^111^In]In-ATH001 (*n* = 20) or [^111^In]In-ATH022 (*n* = 20) (Supplementary Fig. 2). The mice were euthanized at different timepoints after tracer injection, and tumor and tissue uptake assessed by gamma counter as described above. For [^111^In]In-ATH001 the timepoints were 1 h (*n* = 4), 4 h (*n* = 4), 24 h (*n* = 4), 72 h (*n* = 4) and 168 h (*n* = 4). These groups were administered 0.28 µg (40 pmol) of [^111^In]In-ATH001 30 kBq for the 1, 4, and 24 h, and 60 kBq for the 72 and 168 h time points).

For [^111^In]In-ATH022, the timepoints were 1 h (*n* = 8, of which *n* = 4 co-injected with 10 nmol unlabeled ATH001 for blocking), 4 h (*n* = 4), 24 h (*n* = 4) and 120 h (*n* = 4). Mice were here administered 0.55 µg (40 pmol) of [^111^In]In-ATH022 (30 kBq for the 1, 4, and 24 h, and 60 kBq for the 120 h time points).

### PET/CT imaging in pig

A direct comparison of the biodistribution of [^68^Ga]Ga-ATH001 and [^68^Ga]Ga-ATH022 was conducted in a pig, which has a physiology and size more similar to humans compared to mice.

A Swedish Landrace pig (male, 8 weeks old, 33 kg) was anaesthetized as described in the Supplementary Materials and intravenous catheters were placed for tracer injection. The pig was positioned supine within the gantry of a PET/CT scanner (Discovery MI, GE Healthcare, 25 cm Field of View). A whole body CT scan was performed to facilitate attenuation correction and anatomical alignment of the PET data.

1 MBq/kg [^68^Ga]Ga-ATH001 (corresponding to 1 µg/kg peptide, 33 µg, 4.7 nmol) was administered as a bolus and the following PET sequence was started at injection: first a 15-min dynamic scan of the heart, followed by a series of whole-body passes covering the brain to the bladder—in total 9 passes over 170 min.

Next, at least 3 h were allowed to pass for the radioactivity from the first injection to excrete and decay. Then, 4 MBq/kg [^68^Ga]Ga-ATH022 (corresponding to 2 µg/kg peptide, taking into account the higher molar weight of the dimeric construct, 66 µg, 4.8 nmol) was administered intravenously, followed by the same scanning protocol as described above.

### Image analysis of mouse and pig PET data

SUV corrected images were generated for the mouse PET/MRI and pig PET/CT scans, using PMOD 4.0 (PMOD Technologies). For the pig, all major organs and the blood pool (left ventricle) were segmented and the uptake over time expressed as Standard Uptake Values (SUV).

### Statistics

Data was visualized and analyzed using GraphPad Prism 10. Differences between groups were analyzed using Students T-test or, for multigroup analysis, ANOVA.

## Results

### SPR assay for affinity towards PDGFRβ

**Fig. 2:**
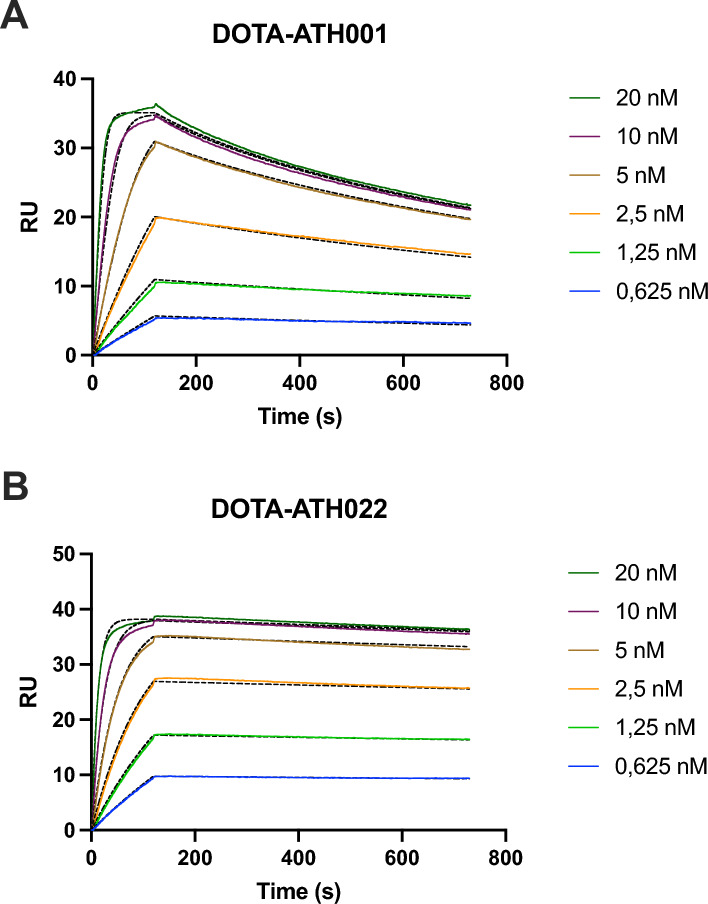
SPR sensorgrams for the interaction between PDGFRβ-Fc (ligand) and, respectively, **A** DOTA-ATH001 monomer (analyte) and **B** dimer DOTA-ATH022 (analyte). Dotted lines indicate the 1:1 interaction model fitting for kinetic parameter determination

To quantify the avidity arising from dimerization, the interaction between NHS-immobilized PDGFRβ-Fc and either DOTA-ATH001 or DOTA-ATH022 was investigated using surface plasmon resonance (SPR). A marked increase in apparent affinity was observed for the dimer DOTA-ATH022, which displayed almost a ten times lower equilibrium dissociation constant than its monomeric counterpart DOTA-ATH001 (19 ± 5 pM vs. 160 ± 20 pM) (Fig. 2 and Table 1). As could be expected for an avidity-driven increase in apparent affinity, this improvement was due to a slower dissociation. Dimerization decreased the off-rate of the interaction by approximately an order of magnitude whereas the on-rate, while also slower, was much less affected. The observed slower dissociation for DOTA-ATH022 provides a strong indication that both Affibody molecule moieties of this dimer can interact with separate PDGFRβ molecules simultaneously.

**Table 1:** The kinetic parameters and equilibrium dissociation constants (K_D_) for the interaction between PDGFRβ-Fc and, respectively, DOTA-ATH001 and DOTA-ATH022, as determined by SPR

Analyte	K_D_ (M)	k_on_ (M^−1^ s^−1^)	k_off_ (s^−1^)
DOTA-ATH001	(1.6 ± 0.23)*10^–10^	(7.1 ± 0.53)*10^6^	(1.2 ± 0.16)*10^–3^
DOTA-ATH022	(1.9 ± 0.46)*10^–11^	(4.6 ± 0.39)*10^6^	(8.7 ± 0.27)*10^–5^

### PDGFRβ activation assay

**Fig. 3:**
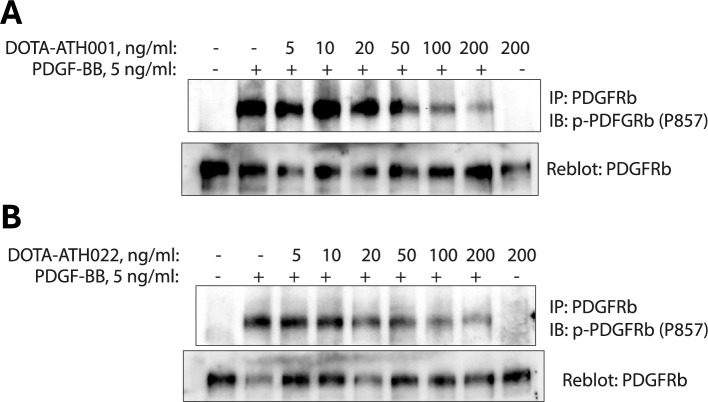
**A** In vitro activation assay showing that DOTA-ATH001 is a PDGFRβ antagonist. PDGFRβ was immunoprecipitated from LX-2 cells stimulated with PDGF-BB and treated with monomeric DOTA-ATH001, as indicated. Activation of PDGFRβ was detected by immunoblotting with a phospho-tyrosine 857-specific antibody. The presence of the immunoprecipitated receptor in all samples was confirmed by reblotting the membrane with the antibody against total PDGFRβ (bottom panel). **B** The dimerized construct DOTA-ATH022 also acted as competitive antagonist towards PDGFRβ in LX-2 cells. The experiment was conducted as described in (**A**)

In order to evaluate the ability of the dimeric DOTA-ATH022 to interfere with the activation of PDGFRβ, we conducted a PDGFRβ activation assay whereby LX-2 cells were stimulated with 5 ng/ml PDGF-BB ligand for 20 min with or without increasing concentrations of the Affibody molecule. The readout of the activation of PDGFRβ was PDGF-BB-induced phosphorylation of the receptor as determined with an antibody against phosphorylated tyrosine 857 (P857) that is positioned in the activation loop of the receptor. We found that dimeric DOTA-ATH022 inhibited activation of PDGFRβ at concentrations higher than 20 ng/ml (Fig. 3B) with equal efficiency as the monomeric DOTA-ATH001 (Fig. 3A). We also found that dimeric DOTA-ATH022 was not able to activate the receptor by binding to it in the absence of PDGF-BB, as no activation was detected after treatment with 200 ng/ml of DOTA-ATH022.

### Gallium-68 and Indium-111 radiolabeling of DOTA-ATH001 and DOTA-ATH022

[^68^Ga]Ga-ATH022, [^68^Ga]Ga-ATH001, [^111^In]In-ATH022 and [^111^In]In-ATH001 was consistently produced with a purity in the range of 95% (Supplementary Fig. 3A–D). The tracers were formulated in PBS with an apparent molar activity in the range of 10–20 MBq/nmol for each delivery, to enable all planned in vitro and in vivo experiments.

### Lipophilicity assay

Dimeric [^68^Ga]Ga-ATH022 (LogD_7.4_ = − 1.74) was slightly more lipophilic than monomeric [^68^Ga]Ga-ATH001 (LogD_7.4_ = − 1.30) at biological pH 7.4.

### Plasma stability of [^68^Ga]Ga-ATH001 and [.^68^Ga]Ga-ATH022

The stability of [⁶⁸Ga]Ga-ATH001 and [⁶⁸Ga]Ga-ATH022 was assessed following 60 min incubation in human plasma at 37 °C. The samples were analyzed for total precipitate formation, the activity retained in the supernatant, and the radiochemical purity (RCP) using HPLC (Supplementary Table 1). For the [^68^Ga]Ga-ATH001, the average precipitate formation was 21 ± 1%, with 79 ± 1% of activity retained in the supernatant. HPLC analysis revealed an average RCP of 82 ± 2% (*n* = 3) in the human plasma. In contrast, the [^68^Ga]Ga-ATH022 showed precipitate formation of 25 ± 6%, with 75 ± 6% of activity remaining in the supernatant. The dimer demonstrated an average RCP of 88% (*n* = 2) in the human plasma, i.e. almost 90% intact tracer after 60 min incubation.

### In vitro binding and retention to U87 and LX-2 cells

**Fig. 4:**
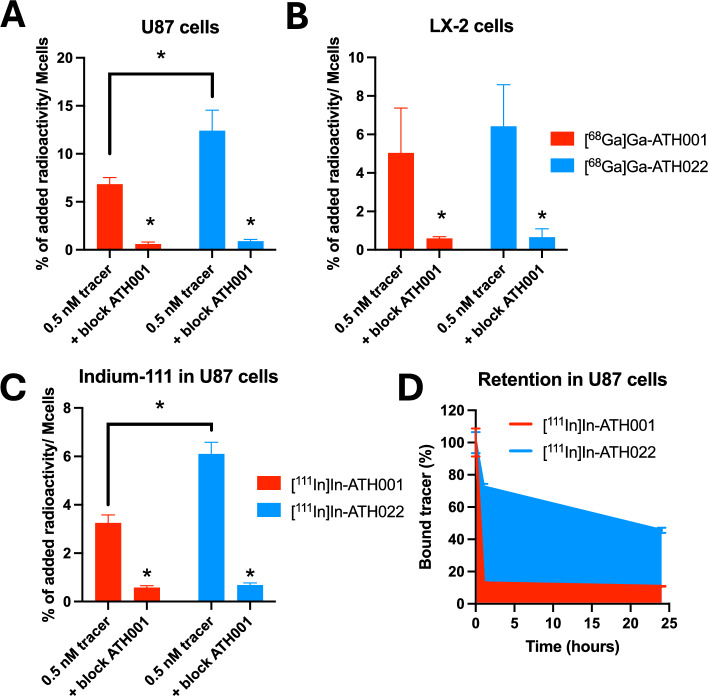
In vitro cell specificity assay of [^68^Ga]Ga-ATH001 (red bars) and [^68^Ga]Ga-ATH022 (blue bars) in U87 (**A**) and LX-2 cells (**B**). In vitro cell specificity assay for [^111^In]In-ATH001 or [^111^In]In-ATH022 in U87 cells (**C**). Cell retention assay monitoring the U87 cell binding of [^111^In]In-ATH001 and [^111^In]In-ATH022 for up to 24 h (**D**). *p < 0.05

In vitro binding specificity of [^68^Ga]Ga-ATH022 and [^68^Ga]Ga-ATH001 was assessed by a binding specificity assay using PDGFRβ expressing U87 and LX-2 cells. The binding was significantly decreased for both tracers in both cell lines when the receptor was pre-saturated by unlabelled ATH001, demonstrating binding specificity (Fig. 4A-B). Furthermore, [^68^Ga]Ga-ATH022 demonstrated higher binding to U87 cells than [^68^Ga]Ga-ATH001 at equal concentrations (12.4 ± 2.1 vs 6.8 ± 0.7 percent of added radioactivity per million cells) (Fig. 4A).

Similarly, [^111^In]In-ATH022 exhibited higher specific binding to U87, compared to [^111^In]In-ATH001 (Fig. 4C). [^111^In]In-ATH022 also demonstrated a much higher retention in U87 cells, with more than 40% bound remaining after 24 h compared to around 10% for [^111^In]In-ATH001 (Fig. 4D). This is presumably due to the same avidity effects seen in the SPR assay, where the two binders in the dimeric construct interacted with separate PDGFRβ receptors present on the cell surface.

### In vitro LigandTracer assay

In vitro binding kinetics and affinity of [^111^In]In-ATH001 and [^111^In]In-ATH022 were measured in real-time by a LigandTracer assay. Representative association and dissociation curves are shown in Supplementary Fig. 4A, B and the kinetic evaluations are presented in Table 2. [^111^In]In-ATH022 demonstrated a similar association (on-rate) as [^111^In]In-ATH001, while the dissociation (off-rate) was decreased with almost a magnitude ((0.45 ± 0.18)*10^–5^ vs (5.5 ± 4.7)*10^–5^) (Supplementary Fig. 4C, D and Table 2). The estimated affinity of [^111^In]In-ATH022 (off-rate divided by on-rate) was approximately 10–20 times improved compared to [^111^In]In-ATH001 (6.0 ± 1.2 pM vs. 140 ± 120 pM) (Supplementary Fig. 4E and Table 2), mainly driven by the much slower off-rate (representative comparison in Supplementary Fig. 4F), presumably due to avidity affects due to the dimeric design of ATH022.

**Table 2:** Kinetic parameters and equilibrium dissociation constants (K_D_) for the interaction between [^111^In]In-ATH001 or [^111^In]In-ATH022 and PDGFRβ on U87 cells, as determined by the LigandTracer assay

Analyte	K_D_ (M)	k_on_ (M^−1^ s^−1^)	k_off_ (s^−1^)
[^111^In]In-ATH001	(140 ± 120)*10^–12^	(4.9 ± 2.9)*10^5^	(5.5 ± 4.7)*10^–5^
[^111^In]In-ATH022	(6.0 ± 1.2)*10^–12^	(7.3 ± 1.8)*10^5^	(0.45 ± 0.18)*10^–5^

### Biodistribution of [^68^Ga]Ga-ATH022 and [^68^Ga]Ga-ATH001 in U87 xenografted mice

**Fig. 5:**
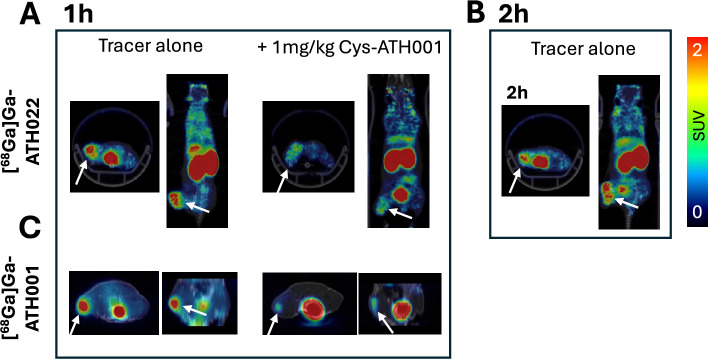
Representative PET images demonstrating binding of [^68^Ga]Ga-ATH022 at 1 h (**A**) or 2 h (**B**), and [^68^Ga]Ga-ATH001 1 h after injection (**C**) in U87 xenograft tumors (white arrows). Blocked binding after co-injection of unlabeled ATH001 is also shown (**A**) and (**C**)

**Fig. 6:**
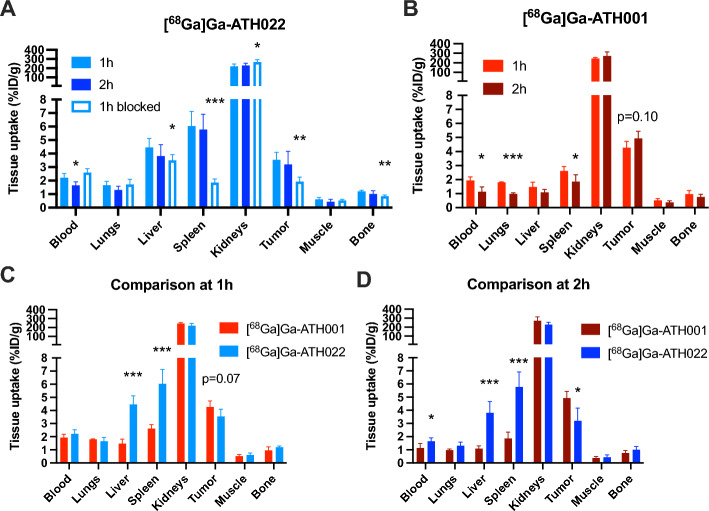
In vivo biodistribution and tissue binding of [^68^Ga]Ga-ATH001 (red bars, **A**) and [^68^Ga]Ga-ATH022 (blue bars, **B**) in U87 xenografted immunodeficient mice. Open bar in panle B indicate in vivo blocking of [^68^Ga]Ga-ATH022. Direct comparison of biodistribution of [^68^Ga]Ga-ATH001 and [^68^Ga]Ga-ATH022 after 1 h (**C**) or 2 h (**D**). Stars (*) indicate difference compared to [^68^Ga]Ga-ATH001. *p < 0.05, **p < 0.01, ***p < 0.001

[^68^Ga]Ga-ATH022 demonstrated rapid distribution, tissue targeting and clearance despite its increased size compared to [^68^Ga]Ga-ATH001 (Fig. 5A-B, Fig. 6A and Supplementary Fig. 5). Strong, blockable binding was seen in U87 tumors and spleen, tissues with expression of PDGFRβ protein as shown by immunohistochemistry (Supplementary Fig. 6A-C). There was also a slight increase in blockable binding in liver. Tissue binding was retained after 2 h, while blood radioactivity decreased, likely due to renal excretion given the high kidney uptake (Fig. 5B and Fig. 6A). Low binding was seen in PDGFRβ-negative tissues such as muscle (Fig. 6A and Supplementary Fig. 6A–C). [^68^Ga]Ga-ATH001, by contrast, demonstrated somewhat more rapid blood clearance, with retained tumor binding at 2 h post-injection, but also lower retention in PDGFRβ positive spleen (Fig. 5C and Fig. 6B).

[^68^Ga]Ga-ATH022 exhibited up to 3 times higher binding than [^68^Ga]Ga-ATH001 in the well perfused spleen at the two hour time-point (5.8 ± 1.1 vs. 1.8 ± 0.5%ID/g) (Supplementary Table 2 and Fig. 6C-D), in line with the strong presence of PDGFRβ in this tissue, and the higher affinity of the dimeric construct demonstrated in vitro. On the other hand, [^68^Ga]Ga-ATH022 showed lower uptake in the U87 tumor after 2 h compared to [^68^Ga]Ga-ATH001 (3.2 ± 1.0 vs. 4.9 ± 0.5%ID/g) (Fig. 6D), perhaps due to the dense tumor microenvironment restricting tissue penetration of larger peptide constructs.

The observations above remained when correcting for background activity in circulation (tissue-to-blood-ratio) (Supplementary Fig. 7).

### Biodistribution of [^111^In]In-ATH022 and [^111^In]In-ATH001 in U87 xenografted mice

The in vitro analysis of DOTA-ATH022 indicated that the improved affinity compared to DOTA-ATH001 was due to a strong decrease in off-rate kinetics leading to time-on-target of > 8 h. Thus, it can be hypothesized that the binding retention of DOTA-ATH022 in PDGFRβ positive tissues would be higher also in vivo for hours or days after administration. Thus, we performed a biodistribution study in U87 xenografted mice for up to one week using Indium-111 labeled DOTA-ATH022 and DOTA-ATH001.

**Fig. 7:**
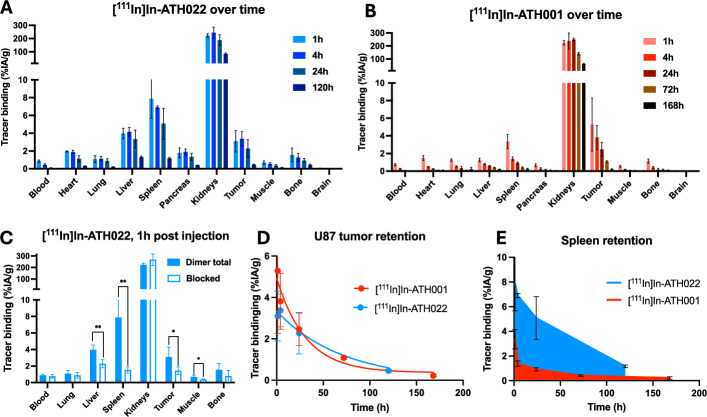
Ex vivo evaluation of biodistribtion using [^111^In]In-ATH022 (blue bars, **A**) and [^111^In]In-ATH001 (red bars, **B**) to investigate the retention in tissues for up to 7 days. In vivo blocking of [^111^In]In-ATH022 at 1 h post injection demonstrating specificity **C**. Retention of [^111^In]In-ATH022 (blue circles) and [^111^In]In-ATH001 (red circles) in U87 tumors (**D**). Retention of [^111^In]In-ATH022 (blue) and [^111^In]In-ATH001 (red) in PDGFRβ-positive spleen (**E**). *p < 0.05, **p < 0.01

[^111^In]In-ATH022 demonstrated a similar binding profile as [^111^In]In-ATH001 for the first hour, with binding to PDGFRβ positive tissues spleen (7.9 ± 2.2%ID/g) and U87 tumor (3.1 ± 1.2%ID/g) (Fig. 7A). Binding remained high in e.g. tumor at 4 (3.4 ± 0.8%ID/g) and 24 (2.3 ± 1.0%ID/g) hours, as predicted by the slow off-rate seen in vitro. By contrast, [^111^In]In-ATH001 exhibited faster clearance not only from circulation but also spleen and U87 tumor (Fig. 7B).

[^111^In]In-ATH022 binding to spleen and U87 tumor could be blocked by pre-treatment with unlabeled ATH001 (7.9 ± 2.2 vs. 1.5 ± 0.5%ID/g for spleen and 3.1 ± 1.2 vs. 1.4 ± 0.5%ID/g in tumor), similar as for [^68^Ga]Ga-ATH022 (Fig. 7C). The retention of the Indium-111 labeled tracers in U87 tumors was estimated by a mono-exponential fit to the available time-points. [^111^In]In-ATH022 was described by a t_1/2_ = 48.6 h (*K* = 0.014 h^−1^), more than twice that of [^111^In]In-ATH001 (t_1/2_ = 21.8 h, *K* = 0.032 h^−1^) (Fig. 7D), although [^111^In]In-ATH001 exhibited higher initial uptake. The increased retention in PDGFRβ avid spleen was even greater: t_1/2_ = 34.8 h (*K* = 0.020 h^−1^) for [^111^In]In-ATH022 compared to t_1/2_ = 1.8 h (*K* = 0.40 h^−1^) for [^111^In]In-ATH001 (Fig. 7E), and an area under the curve almost 5 times higher.

### Biodistribution in pig

[^68^Ga]Ga-ATH001 demonstrated a rapid biodistribution and clearance also in pig, with low background accumulation in most tissues, except kidney, already after 1-h post-injection (Supplementary Fig. 8A, C). Spleen binding was low, as opposed to in mice. This may be due to either lower PDGFRβ concentration in pig spleen, or decreased affinity of the Affibody molecule to the porcine receptor. [^68^Ga]Ga-ATH022 on the other hand exhibited slower clearance and washout from all tissues including blood but reached a similar background level as [^68^Ga]Ga-ATH001 after 3 h (Supplementary Fig. 8B, D). The slower clearance is presumably due to the almost double size of [^68^Ga]Ga-ATH022. Kidney retention was also almost twice as high for [^68^Ga]Ga-ATH022 during the course of the scan (Supplementary Fig. 8E). Calculation of the tissue uptake ratio between dimeric ATH022 and monomeric ATH001 reinforced the notion that [^68^Ga]Ga-ATH022 displayed slower tissue clearance, especially kidney and liver, but otherwise normalized after around 3 h after administration (Supplementary Fig. 8F).

## Discussion

Affinity to its intended target is one of the most crucial properties that determines the performance of radiopharmaceuticals (Adams et al. 1998; Xenaki et al. 2017). Here we applied a dimerization approach to improve the affinity using a novel tri-function linker to enable a construct comprising two binding molecules as well as a DOTA chelator. The linker is amenable to facile coupling of any type of peptide binders, not only Affibody molecules, and can potentially combine different binders to either homo- or hetero-dimer for potentially highly specific targeting of well-defined cell populations. The DOTA moiety could also be replaced by other chelators, reporting systems or even therapeutic payloads.

Here, we show proof-of-concept of this dimerization technology using Affibody molecule ATH001—a binder with sub-nanomolar affinity towards PDGFRβ which is currently in clinical development for PET imaging of fibrotic disease in indications including e.g. metabolic dysfunction-associated steatohepatitis (MASH) (NCT06562361) and cardiac fibrosis (NCT06956560) (Yashaswini et al. 2025). The homo-dimeric construct DOTA-ATH022 was generated by chemical synthesis enabling high purity while excluding the need for e.g. His-tags that may adversely affect biodistribution.

Affinity can be improved by molecular redesign which may cost both time and resources. Homo-dimerization, on the other hand, uses existing binders while exploiting the avidity phenomena, e.g. that multiple individual interactions synergistically contribute to the total binding strength. This approach can be especially powerful when the target itself can homo-dimerize, which is the case for PDGFRβ. We thus hypothesized that the presence of several PDGFRβ molecules in close proximity on the cell surface would be readily available for ATH022 binding, enabling strong avidity effects compared to the binding of ATH001 to a single PDGFRβ molecule.

Longer time on target is important for radiopharmaceuticals to allow for clearance from blood circulation and tissues and lower background signal. This is true not least for optimal image contrast from PET tracers, but is also critical for therapeutic radionuclides, given the dosimetry considerations (target tissue vs healthy tissues). Increased affinity, especially driven by decreased off-rate, theoretically leads to longer retention at the target receptor.

Here, we demonstrate that dimerization of ATH001 into the construct ATH022 results in a strong improvement in affinity towards PDGFRβ—up to 8 times by SPR and up to 10–20 times by U87 cell binding using the LigandTracer technology. In both assays, the improvement was primarily due to a significantly lower off-rate of the binding interaction, i.e. the ATH022 construct dissociates more slowly from the receptor(s).

DOTA-ATH022 acted as a competitive antagonist and blocked PDGF-BB mediated activation of PDGFRβ on the cell surface dose-dependently. The potency was similar to that of DOTA-ATH001 and here the dimerization of the construct did not seem to have any stronger effects. DOTA-ATH022 itself did not induce activation of PDGFRβ and therefore was not able to promote a formation of a functional PDGFRβ homodimer.

DOTA-ATH022 labeled with either Gallium-68 or Indium-111 readily targeted PDGFRβ avid tissues also in vivo. In spleen for example, ATH022 exhibited stronger retention after binding, resulting in a long-term higher exposure than the monomeric counterpart ATH001. Both tracers were stable in plasma and thus differences in binding in vivo are expected to be due mainly to affinity.

However, the uptake in solid U87 tumors immediately after tracer administration was lower for ATH022 than for ATH001. This is potentially due to the larger molecular size of DOTA-ATH022 (13.8 kDa) compared to DOTA-ATH001 (7.1 kDa). Monomeric Affibody molecules with a relatively small size of ≈6.5–7.5 kDa have excellent tissue penetration properties, including in dense tumor interstitium (Schmidt and Wittrup 2009). On the other hand, larger proteins and especially antibodies may exhibit poor penetration in solid tumors with high interstitial fluid pressure (IFP) (Heldin et al. 2004). The dimeric construct DOTA-ATH022 is twice as large as DOTA-ATH001, and share a size similar to that of e.g. single domain antibodies (sdAb) (also referred to as Variable regions of the Heavy chain of Heavy-chain only immunoglobulins (VHH) or trademarked as Nanobodies, in the range of 12–15 kDa). The experimental results presented here indicates that monomeric Affibody molecules demonstrated superior tumor tissue penetration in vivo, compared to dimeric constructs with a size in the range of sdAbs, even when the larger homo-dimer exhibited much better affinity. Thus, even approximately 10 times improved affinity here could not overcome the decreased tissue penetration of the larger molecule.

Additionally, the strongly increased uptake of DOTA-ATH022 in spleen and liver (2–3 times higher than for DOTA-ATH001 at all time points) may also cause a “sink effect” (Fig. 6C-D). A substantially increased first pass hepatic and splenic accumulation of radiolabeled DOTA-ATH022 could potentially limit the fraction available for tumor uptake. On the other hand, there is no clear reduction in blood concentration of DOTA-ATH022 which indicates that the sink effect is moderate.

Finally, another potential reason for the modest initial in vivo tumor uptake of ATH022 may be the binding-site barrier (BSB) effect (Miao et al. 2016; Qvarnström et al. 2011; Song et al. 2021). The BSB effect is a phenomenon where high-affinity agents (like antibodies or small proteins) preferentially accumulate in perivascular regions of a solid tumor, limiting their deep penetration into the rest of the tumor tissue. This effect and consequently uneven distribution may compromise the overall tumor uptake of ATH022 to a higher degree than ATH001, given the improved affinity of the former. The BSB effect in the context of ATH022 is merely a suggested mechanism and has not been experimentally investigated. However, assays such as ex vivo autoradiography could potentially be used to directly observe or rule out this effect. Furthermore, additional mechanisms may be responsible for the discrepancy between lack of improved tumor binding and the increased avidity seen here.

The early uptake in other healthy, well-perfused PDGFRβ positive tissues such as spleen, on the other hand, was higher for DOTA-ATH022, correlating reasonably with the improved affinity. This is of special interest when considering PET imaging of pathologies that involve increased tissue perfusion and permeability, such as inflammation. The experimental results indicate that the increased molecular size of the dimeric construct has negligible impact on the uptake and binding in highly permeable tissues, i.e. the negative influence on size seen in solid tumors is not observed. Thus, we hypothesize that the benefit of homo- or heterodimeric Affibody constructs (based on the tri-functional linked used here)—e.g. improved affinity and tissue exposure—can be harnessed in full in indications involving inflammation. This possibility remains to be investigated further.

## Conclusion

DOTA-ATH022 is a novel dimerized version of Affibody molecule ATH001, with significantly improved affinity towards PDGFRβ. Retention of radiolabeled DOTA-ATH022 in PDGFRβ-avid tissues and tumors was increased, however uptake in solid tumor tissue was not improved.

## Supplementary Information

Supplementary material 1

## Data Availability

The datasets used and/or analyzed during the current study are available from the corresponding author on reasonable request.
