# Supplementary material for: Increased affinity by dimerization of radiolabeled Affibody molecule ATH001 targeting PDGFRβ
Source: EJNMMI Radiopharm Chem. 2026 Mar 16;11:25. doi: 10.1186/s41181-026-00439-x (PMC13066081; doi:10.1186/s41181-026-00439-x)
Supplement: Supplementary file 1 — Supplementary material 1 [file 41181_2026_439_MOESM1_ESM.docx]

**Supplementary Data**

**Increased affinity by dimerization of radiolabeled Affibody molecule ATH001 targeting PDGFRβ**

Ayman Abouzayed et al.

**Supplementary Methods**

*Pig anaethesia and monitoring*

The pig was anaestetized and monitored by trained staff using standard techniques, under oversight by a veterinary. The pig was sedated via intramuscular injection of tiletamine-zolazepam (Boehringer Ingelheim), and then the animal was intubated and placed on mechanical ventilation; anesthesia was maintained through continuous intravenous infusion of ketamine (20 mg/kg/h, Ketaminol), fentanyl (5 mg/kg/h, Pharmalink), and pancuronium (0.24 mg/kg/h, Pavulon, Organon Teknika), while under standard physiological monitoring as previously described (1). The pig was allowed to stabilize for 30 minutes and received fluids (500-1000 ml) before transport to the PET scanner.

Following the imaging sessions, the pig was euthanized under deep anesthesia with an intravenous injection of potassium chloride (KCl).

**Supplementary References**

1. Wegrzyniak O, Zhang B, Rokka J, et al. Imaging of fibrogenesis in the liver by [18F]TZ-Z09591, an Affibody molecule targeting platelet derived growth factor receptor β. EJNMMI

**Supplementary Figures**

**Supplementary Figure 1.** Quality control of the DOTA-ATH022 and DOTA-ATH001 peptide precursors. Representative HPLC chromatograms (A-B) and Mass Spectrometry analysis (C-D) are shown.

**Supplementary Figure 2.** Schematic representation of the mice used in the in vivo U87 xenograft experiments in the study.

**Supplementary Figure 3.** Representative HPLC radio-chromatograms for Gallium-68 labeled dimer DOTA-ATH022 (A) and monomer DOTA-ATH001 (B), as well as Indium-111 labeled DOTA-ATH022 (C) and DOTA-ATH001(D).

**Supplementary Figure 4.** Representative kinetic curves from the LigandTracer assay using [^111^In]In-ATH001 (A) or [^111^In]In-ATH022 (B)

**Supplementary Figure 5.** Maximum Intensity Projection (MIP) PET images demonstrating whole-body biodistribution of [^68^Ga]Ga-ATH022 in xenograft-carrying mouse. The image was acquired 2 hours post-injection. Bl = bladder, Ki = kidney, Li = liver, Tu = tumor,

**Supplementary Figure 6.** Representative staining of tissues from U87 xenograft carrying immunodeficient mice used for biodistribution assessment in this study. Spleen was included as positive control and muscle as negative control. Massons Trichrome (MTC) staining for collagen (blue; A), PDGFRβ immunostaining (brown) showing protein expression in the U87 xenograft tumor and in spleen (B), as well as Hematoxylin and eosin (H/E) staining (C), was performed.

**Supplementary Figure 7.** Tissue-to-blood uptake ratio for Gallium-68 labeled ATH022 and ATH001 in U87 xenograft carrying immunodeficient mice. “α” denotes significant increase (p<0.05) in tumor uptake of ATH001 at 2 h compared to 1 h. “#” denotes significant difference (p<0.05) in tissue uptake of ATH022 compared to ATH001 at the same timepoint. * indicate significant blocking effect (p<0.05) on ATH022 tissue uptake.

**Supplementary Figure 8.** Representative coronal images of biodistribution of [^68^Ga]Ga-ATH001 (A) and [^68^Ga]Ga-ATH022 (B) in pig as assessed by in vivo PET/CT imaging. Images are normalized to SUV=5. Tissue uptake over time in major organs quantified as SUV for [^68^Ga]Ga-ATH001 (C) and [^68^Ga]Ga-ATH022 (D). Uptake in kidneys of each tracer expressed as SUV (E). Tissue uptake expressed as ratio calculated as [^68^Ga]Ga-ATH022 SUV divided by [^68^Ga]Ga-ATH001 SUV at each time-point (F).

**Supplementary Table 1.** Comparison of stability of [⁶⁸Ga]Ga-ATH001 and [⁶⁸Ga]Ga-ATH022 in human plasma after 60 minutes incubation in vitro.

| **Tracers** | **[⁶⁸Ga]Ga-ATH001** | **[⁶⁸Ga]Ga-ATH022** |
| --- | --- | --- |
| No of tests | 3 | 2 |
| Total precipitate (%) | 21 ± 1 | 25 ± 6 |
| Supernatant (%) | 79 ± 1 | 75 ± 6 |
| HPLC (%) | 82 ± 2 | 88 ± 0 |

**Supplementary Table 2.** Biodistribution of [^68^Ga]Ga-ATH001 and [^68^Ga]Ga-ATH022 in liver, spleen, kidney and U87 tumor in mice, 1 h or 2 h postinjection. Data presented in %ID/g and averages±SD. * indicates significant increased uptake (p<0.05) compared to the other tracer at the same time-point.

|  | **1 hour** | | **2 hours** | |
| --- | --- | --- | --- | --- |
| **Organs** | **[^68^Ga]Ga-ATH001** | **[^68^Ga]Ga-ATH022** | **[^68^Ga]Ga-ATH001** | **[^68^Ga]Ga-ATH022** |
| Blood | 1.9 ± 0.3 | 2.2 ± 0.3 | 1.1 ± 0.3 | 1.7 ± 0.3 * |
| Liver | 1.5 ± 0.3 | 4.5 ± 0.4 * | 1.1 ± 0.2 | 3.8 ± 0.7 * |
| Spleen | 2.6 ± 0.3 | 6.0 ± 0.3 * | 1.9 ± 0.5 | 5.8 ± 1.1 * |
| Kidney | 244.2 ± 12.6 | 221.0 ± 25.5 | 272.6 ± 42.6 | 228.7± 25.5 |
| Tumor | 4.3 ± 0.4 (p=0.07) | 3.5 ± 0.3 | 4.9 ± 0.5 * | 3.2 ± 0.6 |
